# Perforated Jejunal Diverticulitis Managed Laparoscopically: A Case Report

**DOI:** 10.7759/cureus.82682

**Published:** 2025-04-21

**Authors:** Brenda Santamaria, Diego Viteri Cevallos, Jose Samaniego, Álvaro Morillo Cox, Vicente Peñaherrera, Tatiana Fernandez Trokhimtchouk

**Affiliations:** 1 General Surgery, Universidad Internacional del Ecuador, Quito, ECU; 2 General Surgery, Axxis Hospital, Quito, ECU; 3 General Surgery, Novaclinica, Quito, ECU; 4 Gastroenterology, Axxis Hospital, Quito, ECU; 5 General Surgery, Universidad de las Américas, Quito, ECU

**Keywords:** acute abdomen, diagnostic laparoscopy, jejunal diverticulitis, jejunal diverticulosis, laparoscopic resection, perforated jejunal diverticulum, small bowel perforation

## Abstract

Jejunal diverticulosis is an uncommon condition that is frequently asymptomatic but may present with serious complications such as diverticulitis and, more rarely, perforation. Its clinical presentation is typically nonspecific and often mimics more common abdominal emergencies, which can delay diagnosis and treatment. We report the case of a 63-year-old woman who presented with diffuse abdominal pain, vomiting, and absence of flatus. Computed tomography revealed jejunal wall thickening, multiple diverticula, mesenteric fat stranding, and localized pneumoperitoneum. Diagnostic laparoscopy identified a perforated jejunal diverticulum approximately 20 cm distal to the ligament of Treitz. A 20-cm jejunal segment containing multiple inflamed diverticula was resected, and a primary stapled side-to-side anastomosis was performed. The postoperative course was uneventful, and the patient was discharged on postoperative day four. Histopathology confirmed jejunal diverticulosis with associated inflammation. This case highlights the diagnostic challenges of jejunal diverticulitis and supports the role of individualized, context-specific surgical decision-making. Minimally invasive resection may be a safe and effective approach in select patients with localized perforation.

## Introduction

Jejunal diverticulosis is a rare form of small bowel pathology, with reported prevalence ranging from 0.3% to 6% depending on the population studied and the diagnostic method employed [1-3]. These diverticula are typically acquired pseudodiverticula, formed by herniation of the mucosa and submucosa through weaknesses in the muscularis layer at the entry points of the vasa recta, most commonly along the mesenteric border [4,5]. Their pathogenesis has been linked to abnormal motility, increased intraluminal pressure, and age-related changes in the intestinal wall [6,7].

Although most patients with jejunal diverticula remain asymptomatic, they can occasionally present with vague gastrointestinal symptoms such as bloating, abdominal discomfort, or malabsorption [8,9]. The condition is often underdiagnosed, especially in elderly patients, and its complications, such as diverticulitis, hemorrhage, or perforation, can be life-threatening and require prompt intervention [10,11].

Diagnosis is challenging due to the nonspecific nature of symptoms and the rarity of the disease. In many cases, especially during acute presentations, the condition mimics more common pathologies such as colonic diverticulitis, perforated peptic ulcer, or small bowel obstruction. Cross-sectional imaging with contrast-enhanced computed tomography (CT) plays a pivotal role in diagnosis, although jejunal diverticulitis may be missed or misinterpreted in up to 50% of cases [4,5].

Given its low incidence and non-pathognomonic clinical presentation, jejunal diverticulitis remains a diagnostic challenge. Here, we present a case of perforated jejunal diverticulitis in a 63-year-old woman with known colonic diverticulosis, successfully managed by laparoscopic segmental resection. This case adds to the growing body of literature emphasizing the need for early imaging and tailored surgical management in complicated jejunal diverticulitis.

## Case presentation

We report the case of a 63-year-old female with a medical history of arterial hypertension, ventricular extrasystoles, hypothyroidism, and prior open appendectomy. She presented to the emergency department with a 12-hour history of diffuse, moderate-intensity abdominal pain without clear precipitating factors. The pain was accompanied by two episodes of non-bilious vomiting and absence of flatus. Due to the progressive worsening of symptoms, she sought medical attention.

On arrival, she was hemodynamically stable, with blood pressure of 103/70 mmHg, heart rate of 88 bpm, respiratory rate of 18 breaths per minute, temperature of 36.6°C, and oxygen saturation of 91% on room air. Her body mass index was 31.68 kg/m² (class I obesity). Physical examination revealed moderate abdominal distension, hypoactive bowel sounds, and diffuse abdominal tenderness, more pronounced in the left upper quadrant, with associated rigidity and rebound tenderness.

**Figure 1:**
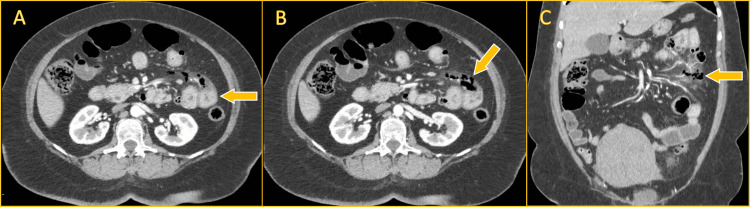
Contrast-enhanced abdominal CT demonstrating findings consistent with complicated jejunal diverticulitis (A) Axial view showing segmental wall thickening of a jejunal loop with multiple diverticula (arrow), indicative of an inflamed segment. (B) Axial view demonstrating adjacent extraluminal gas (arrow) and mesenteric fat stranding, consistent with localized perforation. (C) Coronal view confirming the presence of extraluminal air (arrow) adjacent to the affected jejunal segment, along with surrounding inflammatory changes. An incidental large uterine myoma is also visible in the pelvis.

Laboratory evaluation revealed leukocytosis with neutrophilia and no significant electrolyte abnormalities. Initial laboratory values are summarized in Table 1. Given the patient's clinical presentation, particularly diffuse abdominal pain predominantly in the left abdomen, initial differential diagnoses included acute colonic diverticulitis complicated by perforation or abscess formation, perforated peptic ulcer, and bowel obstruction secondary to adhesions. Imaging was therefore essential to clarify the precise diagnosis. A contrast-enhanced CT scan of the abdomen and pelvis demonstrated segmental wall thickening of the jejunum with multiple diverticula, adjacent mesenteric fat stranding, surrounding laminar free fluid, and extraluminal air bubbles near the affected segment - findings consistent with localized pneumoperitoneum. These imaging features raised suspicion for a perforated jejunal diverticulum (Figure 1).

**Table 1:** Initial laboratory results obtained upon presentation Values are reported with corresponding reference ranges. Abbreviations: ALT – alanine aminotransferase (TGP); AST – aspartate aminotransferase (TGO); GGT – gamma-glutamyl transferase; AF – alkaline phosphatase; BT – total bilirubin; BD – direct bilirubin; BI – indirect bilirubin; Na – sodium; K – potassium; Cl – chloride. All units are reported in standard SI format.

Parameter	Result	Reference range
Leukocytes	16,930 /µL	4,000–10,000 /µL
Neutrophils	15,186 /µL (89.7%)	2,000–7,500 /µL (40–70%)
Lymphocytes	1,033 /µL (6.1%)	1,000–4,800 /µL (20–45%)
Creatinine	0.78 mg/dL	0.6–1.3 mg/dL
Urea	28.2 mg/dL	15–40 mg/dL
AF	148 U/L	40–130 U/L
GGT	29 U/L	8–61 U/L
Lipase	23 U/L	10–140 U/L
Amylase	72 U/L	30–110 U/L
ALT (TGP)	19 U/L	7–56 U/L
AST (TGO)	18 U/L	5–40 U/L
BT	0.95 mg/dL	0.1–1.2 mg/dL
BD	0.28 mg/dL	0.0–0.3 mg/dL
BI	0.67 mg/dL	0.2–0.9 mg/dL
Glucose	176 mg/dL	70–110 mg/dL
Na	137 mmol/L	135–145 mmol/L
K	4.20 mmol/L	3.5–5.1 mmol/L
Cl	101 mmol/L	98–107 mmol/L

**Figure 2:**
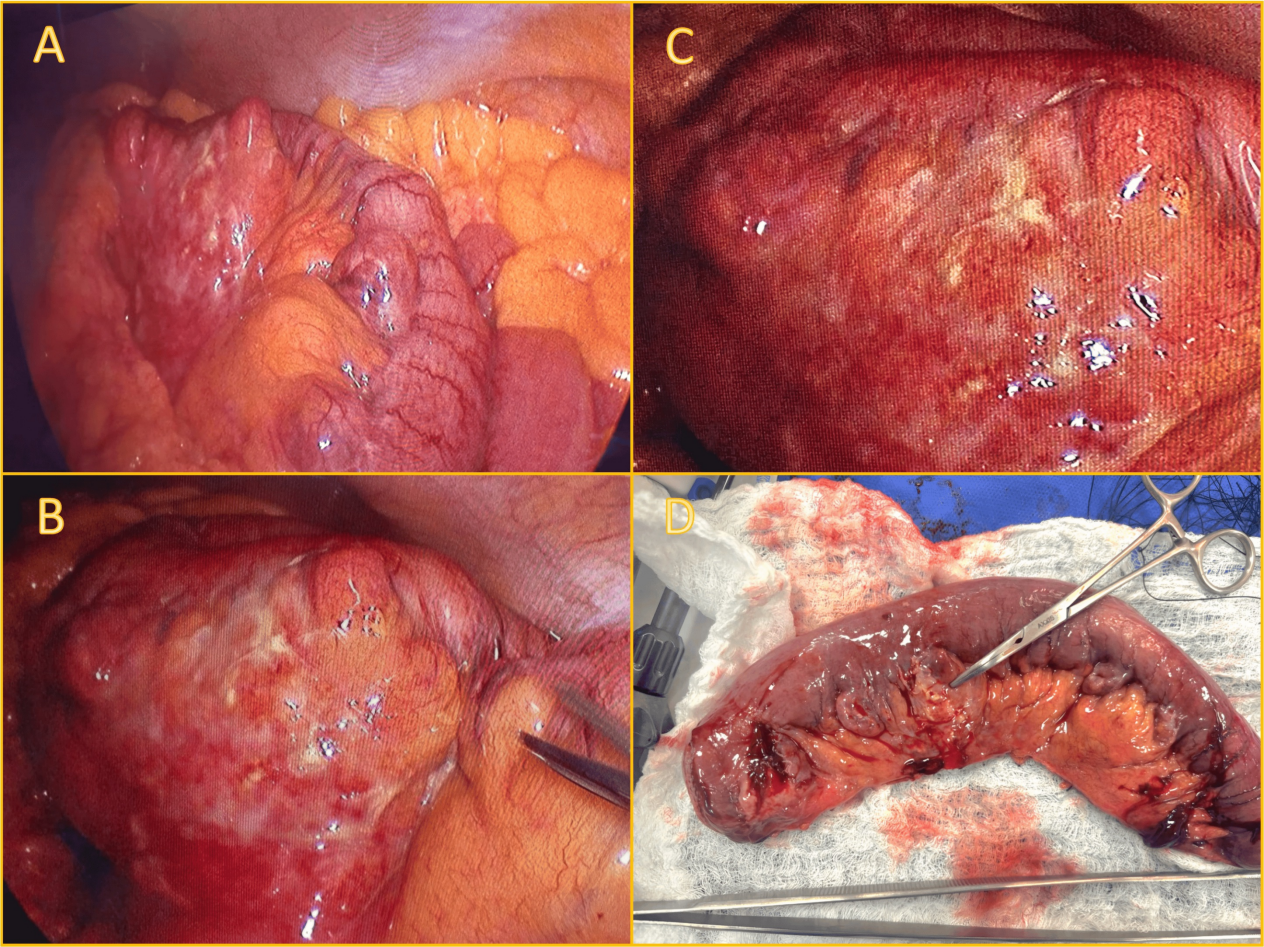
Intraoperative and specimen findings of perforated jejunal diverticulitis (A) Laparoscopic view of the affected jejunal segment showing multiple diverticula with prominent inflammatory changes and fibrinopurulent exudate on the serosal surface. (B) Closer view of the inflamed segment with more pronounced serosal fibrin deposits and reactive mesenteric changes. (C) Detailed laparoscopic view of the perforated diverticulum, with surrounding inflammatory reaction. (D) Resected jejunal specimen displaying multiple diverticula along the mesenteric border. The tip of the Kelly clamp indicates the site of perforation.

The patient was taken emergently to the operating room for diagnostic laparoscopy. Intraoperatively, the small bowel was examined from the ligament of Treitz, revealing a perforated diverticulum approximately 20 cm distal to this landmark. The affected segment exhibited multiple diverticula near the mesenteric border. The perforation site was walled off by surrounding mesentery and omentum, with minimal inflammatory fluid in the cavity. A 20-cm jejunal segment containing the diverticula was resected, and a functional side-to-side anastomosis was created using a 60-mm linear stapler. Relevant images are shown in Figure 2.

The patient received empiric intravenous ampicillin-sulbactam starting preoperatively, which was continued for four days postoperatively. No intraoperative cultures were obtained due to localized inflammation and absence of gross contamination. The antibiotic regimen was transitioned to oral sultamicillin at discharge to complete a total of seven days. No abdominal drains were placed. Postoperative recovery was uneventful, with satisfactory pain control, rapid return of bowel function, and adequate oral tolerance. Histopathologic examination of the resected specimen confirmed jejunal diverticulosis with mucosal ulceration, transmural acute inflammation, focal necrosis, and serosal fibrin deposition - findings consistent with localized perforated diverticulitis.

## Discussion

Jejunal diverticulitis is a rare cause of acute abdomen and often poses significant diagnostic and therapeutic challenges due to its nonspecific presentation and low clinical suspicion. In the absence of pathognomonic signs, its symptoms may mimic more common abdominal pathologies such as appendicitis, colonic diverticulitis, or peptic ulcer perforation, leading to delayed or missed diagnosis [2,6]. In our patient, the presence of colonic diverticulosis and localized peritonism in the left upper quadrant prompted careful evaluation for a small bowel source of sepsis.

While most jejunal diverticula are asymptomatic, complications including inflammation, hemorrhage, obstruction, and perforation can occur in up to 15-30% of cases [4,11]. Perforation, although rare, carries a high mortality rate, reported as high as 40% when diagnosis is delayed [12]. The risk is further exacerbated in elderly patients, in whom vague symptoms and overlapping comorbidities may obscure early recognition.

Contrast-enhanced computed tomography remains the diagnostic modality of choice, but its sensitivity is limited. Small bowel diverticulitis may be missed in up to half of the cases due to subtle findings or misinterpretation, especially when diverticula are not well visualized [4,10]. In our case, CT findings of segmental wall thickening, mesenteric fat stranding, and focal extraluminal air adjacent to jejunal diverticula facilitated timely surgical planning.

Therapeutic strategies depend on the severity of disease and the patient’s clinical status. In stable patients with localized inflammation, conservative management with bowel rest and antibiotics may be considered. Some authors also support CT-guided percutaneous drainage in cases of contained perforation with abscess formation [4,10]. However, failure of nonoperative management and recurrence of symptoms are not uncommon, and delayed surgery may be associated with worsened outcomes.

The empiric antibiotic regimen employed in our patient - intravenous ampicillin-sulbactam transitioned to oral sultamicillin - aligns with current recommendations for community-acquired intra-abdominal infections without diffuse peritonitis or septic shock [13]. However, obtaining intraoperative cultures and targeted microbiological sampling could further enhance antibiotic stewardship by guiding therapy adjustments, especially in cases involving broader contamination, immunocompromised status, or suboptimal clinical improvement.

We advocate for individualized management, based on clinical, radiologic, and intraoperative findings rather than rigid adherence to algorithms. In our patient, despite localized findings on imaging, early laparoscopy was indicated due to signs of systemic inflammation, focal peritonitis, and the presence of multiple inflamed diverticula in a short segment of jejunum. This approach allowed for definitive source control and histopathologic confirmation while minimizing the risks associated with delayed intervention.

Minimally invasive surgery is an evolving standard in emergency abdominal pathology. Although laparotomy is frequently employed for jejunal perforation, laparoscopy, when performed by experienced teams, offers diagnostic accuracy, therapeutic efficacy, and postoperative advantages including reduced pain, morbidity, and hospital stay [14]. This case adds to the limited but growing literature supporting laparoscopy for perforated small bowel diverticulitis in stable patients.

The coexistence of colonic diverticulosis in our patient is a finding reported in up to one-third of individuals with jejunal diverticulosis, suggesting a potential shared pathogenesis, such as underlying connective tissue disorders or dysmotility [6,7]. Recognizing this association is important for expanding the diagnostic differential in elderly patients presenting with acute abdominal pain.

Recent case reports continue to underscore diagnostic challenges and the necessity for surgical intervention in perforated jejunal diverticulitis. Gómez-Carrillo et al. reported successful laparoscopic management of a similar case, emphasizing the benefits of minimally invasive techniques, including rapid recovery and reduced morbidity [15]. Similarly, Chiorescu et al. highlighted diagnostic difficulties, stressing the importance of timely surgical exploration in complicated cases due to limitations of preoperative imaging [16].

This case underscores the importance of maintaining a high index of suspicion, utilizing appropriate imaging modalities, and adopting a patient-specific approach to surgical decision-making. In rare but high-risk conditions such as perforated jejunal diverticulitis, individualized management remains critical for achieving optimal outcomes.

## Conclusions

Perforated jejunal diverticulitis is a rare but serious complication of small bowel diverticulosis that poses diagnostic and therapeutic challenges. Early recognition through cross-sectional imaging and prompt surgical management are essential to reduce morbidity and mortality. This case highlights the effectiveness of diagnostic laparoscopy and limited segmental resection in hemodynamically stable patients with localized jejunal perforation and no diffuse peritonitis, suggesting that minimally invasive surgery may be preferred over open intervention in select cases. A high index of suspicion and a multidisciplinary, individualized approach - guided by clinical context rather than rigid algorithms - is crucial to ensure timely and effective intervention in patients presenting with an acute abdomen of unclear etiology.
